# The effects of peripheral anterior synechiae on refractive outcomes after cataract surgery in eyes with primary angle-closure disease

**DOI:** 10.1097/MD.0000000000024673

**Published:** 2021-04-09

**Authors:** Tae-Eun Lee, Chungkwon Yoo, Yong Yeon Kim

**Affiliations:** a Department of Ophthalmology, Jeonbuk National University Medical School and Hospital, Jeonju; b Department of Ophthalmology, Korea University College of Medicine, Seoul, Korea.

**Keywords:** cataract surgery, peripheral anterior synechiae, primary angle-closure, refractive error

## Abstract

Objective of the study was to investigate the effects of peripheral anterior synechiae (PAS) on refractive outcomes after cataract surgery in eyes with primary angle-closure disease (PACD).

This is a retrospective, cross-sectional study. Seventy eyes of 70 PACD patients who underwent phacoemulsification and intraocular lens implantation. Patients were divided into 2 groups based on the presence of PAS on preoperative gonioscopy. The predictive power of the intraocular lens was calculated by the SRK/T, Hoffer Q, Haigis, and Holladay formulae. The mean absolute error (MAE) and predicted refractive errors were compared between PAS (+) and PAS (–) groups. We also evaluated the refractive errors with regards to the extent of PAS in the subanalyses.

The mean MAE was greater in the PAS (+) group with all formulae (0.61–0.70 diopters [D] vs 0.33–0.45 D, all *P* < .05). The eyes with PAS tended towards myopia (−0.30 D to −0.51 D vs −0.05 D to +0.24 D, all *P* < .05). However, the MAEs or predicted refractive errors were not different, irrespective of the extent of PAS in the subanalyses (all, *P* > .05).

The presence or absence of PAS may influence the postoperative refractive outcomes in PACD patients.

## 1 Introduction

Angle-closure is defined by the presence of iridotrabecular contact. Peripheral anterior synechiae (PAS) are permanent adhesions between the iris and the corneoscleral region of the eye.^[1]^ PAS is one of the pathognomonic signs of angle closure and an important sign for classifying the stage of primary angle-closure disease (PACD). The iridotrabecular contact or PAS obstructs the aqueous outflow through the trabecular meshwork, resulting in an increase in intraocular pressure (IOP). Although the mechanism of PAS formation is not entirely clear, PAS is an important risk factor for uncontrolled IOP and primary angle-closure glaucoma.^[2]^

Cataract extraction significantly increases the anterior chamber depth (ACD) in eyes with PACD.^[3–7]^ This anatomical change may be beneficial in lowering IOP, thereby normalizing elevated IOP. Cataract extraction has therefore been suggested as an efficient treatment modality for acute and chronic angle-closure glaucoma.^[3–7]^

However, the intraocular lens (IOL) power predictions in eyes with PACD tend to be less accurate compared with those in nonglaucomatous eyes or glaucomatous eyes with open-angles. Inaccuracy in the IOL power prediction can be caused by a larger capsular volume, loosened lens zonules, or anterior pulling of the lens by the PAS. Unexpected changes in the IOL position induced by postoperative anterior chamber deepening also contribute to refractive errors after cataract extraction.^[8]^ Such unique anatomical conditions of PACD are probably mainly responsible for the greater differences between the predicted refractive error and the actual refractive error after cataract surgery in eyes with PACD.

In the present study, we, therefore, characterized the presence or absence of PAS as a factor that affected the outcomes of refractive error, postcataract surgery in PACD patients.

## 2 Methods

### 2.1 Subjects

We retrospectively reviewed the medical records of patients who had PACD and had undergone uncomplicated phacoemulsification and a single piece acrylic IOL implantation at the Korea University Guro Hospital, Seoul, Republic of Korea, from April 2008 to December 2013. Ethics approval was obtained from the Institutional Review Board of Korea University Guro Hospital. This study adhered to the tenets of the Declaration of Helsinki.

PACD patients included primary angle-closure suspects, and primary angle-closure (PAC) and primary angle-closure glaucoma (PACG) patients. Primary angle-closure suspects were defined as patients with an eye with an occludable angle and an IOP ≤21 mm Hg without PAS or glaucomatous optic neuropathy (GON). PAC was defined as an eye with any degree of PAS or with an occludable angle accompanied by an elevated IOP (>21 mm Hg) and/or iris ischemia (iris whirling and stromal atrophy), but without GON. PACG was defined as an eye with GON in the presence of PAC.^[9]^

Angle status was confirmed by gonioscopy. In each patient, gonioscopy was performed by a single glaucoma specialist (YYK) at presentation or repeated after the clarity of the cornea was restored. The examination was performed at the lowest level of ambient illumination of a slit lamp with a Goldmann-type three mirror lens (OG3MS; Ocular Instruments, Bellevue, WA), avoiding any light passing through the pupil. To distinguish PAS from appositional angle-closure, dynamic gonioscopy was also performed using a four mirror contact lens (Zeiss, Oberkochen, Germany). The direction of a patient's gaze to a certain mirror and exertion of pressure on the cornea were conducted to widen the angle. PAS was considered present when the adhesion reached to the midtrabecular meshwork upon compression gonioscopy. In eyes with PAS, the location and extent of PAS were also recorded. The patients were classified according to the presence (PAS [+] group) or absence of PAS (PAS [–] group) on gonioscopy.

Exclusion criteria included any identifiable ocular pathology that may have induced PAS formation, such as uveitis, iris neovascularisation, and a previous history of trauma or intraocular surgery. Eyes with phacodonesis on slit-lamp examination, phacocomplicated cataract surgery (eg, posterior capsular ruptures), sulcus-fixated IOLs, combined angle surgery (eg, goniosynechialysis), or postoperative complications (eg, uncontrolled IOP spikes or anterior capsular phimosis) were excluded. Eyes with posterior synechiae with the iris adherent to the anterior lens capsule or a small pupil requiring the use of any pupil dilating device during the surgery were also excluded.

### 2.2 Surgical procedures

All cataract surgeries (phacoemulsification and IOL implantations) were performed by a single experienced surgeon (YYK). After topical anesthesia with 0.5% proparacaine hydrochloride (Alcaine; Alcon Laboratories, Fort Worth, TX), a 2.2- or 2.75-mm temporal clear corneal incision was made, and a viscoelastic agent was introduced to maintain the anterior chamber. A continuous curvilinear capsulorrhexis (CCC) was created slightly smaller than the IOL optic size with a bent 26-gauge needle. Phacoemulsification was performed with an Infinity Vision System (Alcon Laboratories). Cortical remnants were removed by irrigation/aspiration, and a foldable acrylic 1-piece IOL was inserted into the capsular bag. The corneal incision was closed with a single 10-0 nylon suture, and the suture was removed at 1 week after surgery.

### 2.3 Data collection and analysis

Preoperative corneal power, axial length (AL), and ACD were measured using an IOL Master (Carl Zeiss Meditec, Jena, Germany). The IOL power was calculated using the SRK/T, Hoffer Q, Haigis, and Holladay formulae. The formula used to select the IOL power was determined by the surgeon for each patient. The refractive error was measured using an automated keratometer (RK-F1; Canon, Tokyo, Japan) at postoperative visits between 1 and 3 months, and the spherical equivalent (SE) was calculated from the measured refractive errors.

The mean absolute error (MAE) was defined as the absolute value of the predicted refractive error. The predicted refractive error was defined as the difference between the actual postoperative SE and the preoperative SE of the refraction predicted by the IOL Master using each formula (predicted refractive error = postoperative SE – preoperative SE of the predicted refraction).

To determine whether the extent of PAS affected the refractive error after cataract surgery, we divided the PAS (+) group into 2 subgroups based on the extent of PAS. The patients with PAS <180° were classified into subgroup 1, and those with PAS ≥180° were classified into subgroup 2.

All statistics were calculated using the Statistical Package for the Social Sciences, version 21.0 (SPSS, Chicago, IL). The independent sample *t*-test was used to compare the differences in the refractive errors between the PAS (+) and PAS (–) groups. Because the data distribution did not show normality in the subanalyses, the Mann–Whitney *U*-test was used to compare the differences in the refractive errors between subgroups. A value of *P* < .05 was considered statistically significant.

## 3 Results

Seventy eyes of 70 patients (59 females) were enrolled in this study. Among them, 43 eyes had PAS and 15 of these had an extensive PAS ≥180°. An Acrysof IQ (SN60WF; Alcon Laboratories) IOL was implanted in 41 eyes, and a Tecnis (ZCB00; Abbott Medical Optics, Santa Ana, CA) IOL was implanted in 29 eyes. Table 1 shows comparisons of the demographics and refractive errors between PAS (+) and PAS (–) groups. There was no significant difference in age, central corneal thickness, AL, ACD, or mean keratometry readings between the PAS (+) and PAS (–) groups (*P* = .789, *P* = .234, *P* = .069, *P* = .498, and *P* = .079, respectively).

**Table 1:** Comparison of demographics and refractive errors of eyes with peripheral anterior synechiae and without peripheral anterior synechiae (values represent the mean ± standard deviation).

Factors	PAS (+)	PAS (−)	
	n = 43	n = 27	*P-*value^∗^
Age, yr	70.1 ± 5.1	69.8 ± 4.6	.789
Sex (male: female)	9: 34	2: 25	.183
CCT, μm	530.7 ± 34.3	541.6 ± 40.9	.234
AL, mm	22.66 ± 0.64	22.38 ± 0.55	.069
ACD, mm	2.42 ± 0.24	2.38 ± 0.25	.498
Mean K, D	44.17 ± 1.65	44.87 ± 1.48	.079
IOL (Acrysof: Tecnis)	25: 18	16: 11	>.99
Mean absolute errors, D			
SRK/T	0.65 ± 0.38	0.45 ± 0.29	.023
Hoffer Q	0.70 ± 0.46	0.34 ± 0.20	<.001
Haigis	0.61 ± 0.43	0.37 ± 0.27	.009
Holladay	0.63 ± 0.41	0.33 ± 0.19	.001
Predicted refractive errors, D			
SRK/T	−0.30 ± 0.70	0.24 ± 0.49	.001
Hoffer Q	−0.51 ± 0.67	−0.05 ± 0.40	.002
Haigis	−0.34 ± 0.67	0.17 ± 0.43	.001
Holladay	−0.42 ± 0.62	0.03 ± 0.39	.001

ACD = anterior chamber depth, AL = axial length, CCT = central corneal thickness, D = diopters, IOL = intraocular lens, K = keratometry, PAS = peripheral anterior synechiae. Acrysof = Acrysof IQ (SN60WF; Alcon, Fort Worth, TX), Tecnis = Tecnis one piece (ZCB00; Abbott Medical Optics, Santa Ana, CA).

∗ The *P-*value using the independent sample *t*-test except for the sex and IOL comparisons (Chi-square test).

In the PAS (+) group, the MAE was significantly larger than in the PAS (–) group using all formulae (SRK/T; *P* = .023, Hoffer Q; *P* < .001, Haigis; *P* = .009, and Holladay: *P* = .001). The refractive error shifts were also significantly different between the 2 groups. The PAS (+) group had a greater degree of myopic shift than that in the PAS (–) group for all formulae (SRK/T; *P* = .001, Hoffer Q; *P* = .002, Haigis; *P* = .001, and Holladay: *P* = .001).

Table 2 lists the demographics and refractive errors in 2 PAS (+) subgroups (PAS <180° and PAS ≥180°). There was no significant difference in age, central corneal thickness, AL, ACD, or mean keratometry between the 2 subgroups (*P* = .888, *P* = .333, *P* = .665, *P* = .949, and *P* = .919, respectively).

**Table 2:** Comparison of demographics and refractive errors depending on the extent of peripheral anterior synechiae (values represent the mean ± standard deviation).

Factors	PAS <180°	PAS ≥180°	*P-*value^∗^
	n = 28	n = 15	
Age, yr	70.3 ± 5.6	69.9 ± 1.4	.888
Sex (male: female)	7: 21	2: 13	.458
CCT, μm	535.1 ± 34.1	522.3 ± 34.2	.333
AL, mm	22.63 ± 0.70	22.73 ± 0.53	.665
ACD, mm	2.41 ± 0.22	2.44 ± 0.26	.949
Mean K, D	44.15 ± 1.76	44.22 ± 1.46	.919
IOL (Acrysof: Tecnis)	17: 11	8: 7	.750
Mean absolute errors, D			
SRK/T	0.65 ± 0.39	0.66 ± 0.37	.656
Hoffer Q	0.72 ± 0.47	0.68 ± 0.45	.929
Haigis	0.62 ± 0.42	0.62 ± 0.45	.959
Holladay	0.64 ± 0.41	0.61 ± 0.42	.740
Predicted refractive errors, D			
SRK/T	−0.22 ± 0.74	−0.44 ± 0.63	.251
Hoffer Q	−0.46 ± 0.73	−0.61 ± 0.54	.422
Haigis	−0.26 ± 0.71	−0.49 ± 0.60	.346
Holladay	−0.38 ± 0.67	−0.52 ± 0.55	.475

ACD = anterior chamber depth, AL = axial length, CCT = central corneal thickness, D = diopters, IOL = intraocular lens, K = keratometry, PAS = peripheral anterior synechiae, Acrysof = Acrysof IQ (SN60WF; Alcon, Fort Worth, TX), Tecnis = Tecnis one piece (ZCB00; Abbott Medical Optics, Santa Ana, CA).

∗ The *P-*value using the Mann–Whitney *U*-test except for the sex and IOL comparisons (Chi-square test).

No significant differences were found in the MAE between the 2 subgroups using all of the formulae (SRK/T; *P* = .656, Hoffer Q; *P* = .929, Haigis; *P* = .959, and Holladay: *P* = .740). Although subgroup 2 tended to have more myopic shift than subgroup 1, there was no significant difference from predicted refractive errors between the 2 subgroups using all of the formulae (SRK/T; *P* = .251, Hoffer Q; *P* = .422, Haigis; *P* = .346, and Holladay: *P* = .475).

Because 2 IOLs were implanted in the study patients, we compared the refractive errors between them. There was no significant difference between Acrysof IQ and Tecnis IOL in MAE (SRK/T; *P* = .341, Hoffer Q; *P* = .976, Haigis; *P* = .689, and Holladay: *P* = .977) and predicted refractive error (SRK/T; *P* = .102, Hoffer Q; *P* = .414, Haigis; *P* = .270, and Holladay: *P* = .166) (Table 3).

**Table 3:** Comparison of refractive errors between Acrysof and Tecnis intraocular lenses (values represent the mean ± standard deviation).

	Acrysof	Tecnis	
	n = 41	n = 29	*P-*value^∗^
Mean absolute errors, D			
SRK/T	0.61 ± 0.39	0.53 ± 0.32	.341
Hoffer Q	0.57 ± 0.79	0.56 ± 0.30	.976
Haigis	0.54 ± 0.42	0.50 ± 0.35	.689
Holladay	0.51 ± 0.43	0.51 ± 0.29	.977
Predicted refractive errors, D			
SRK/T	−0.01 ± 0.73	−0.30 ± 0.54	.102
Hoffer Q	−0.29 ± 0.70	−0.41 ± 0.50	.414
Haigis	−0.07 ± 0.68	−0.24 ± 0.56	.270
Holladay	−0.17 ± 0.65	−0.36 ± 0.46	.166

D = diopters. Acrysof = Acrysof IQ (SN60WF; Alcon, Fort Worth, TX), Tecnis = Tecnis one piece (ZCB00; Abbott Medical Optics, Santa Ana, CA).

∗ The *P-*value using the independent sample *t*-test.

## 4 Discussion

The present study found that IOL power prediction was less accurate in PACD eyes with PAS than in those without PAS. Compared with the PACD eyes without PAS, those with PAS showed more myopic outcomes following uncomplicated phacoemulsification and IOL implantation. However, the postoperative refractive errors were not significantly different between eyes with moderate (PAS <180°) versus severe (PAS ≥180°) extents of PAS in the subanalyses. To our knowledge, this was the first study to report the effect of PAS on the inaccuracy of IOL power predictions in eyes with PACD.

Compared to normal eyes, eyes with angle closure presented the following ocular biometric features: shorter AL, shallower ACD, greater lens thickness, a more anterior lens position, and smaller radius of the anterior and posterior corneal curvature.^[10–21]^ In addition, eyes with PACD often had large intracapsular volumes and looser zonules.^[11,18,22]^ These structural characteristics often induced not only anterior chamber angle crowding but also led to the inaccuracy of IOL power predictions. After cataract extraction, angle crowding may improve with deepening of the anterior chamber along with posterior shifting of the capsular bag. Such posterior displacement of the IOL position and a decrease in AL caused by IOP reduction after cataract surgery may cause a hyperopic shift in IOL power. However, a myopic shift has also been shown to occur as often as a hyperopic shift after cataract surgery in eyes with angle-closure glaucoma.^[8]^

**Figure 1:**
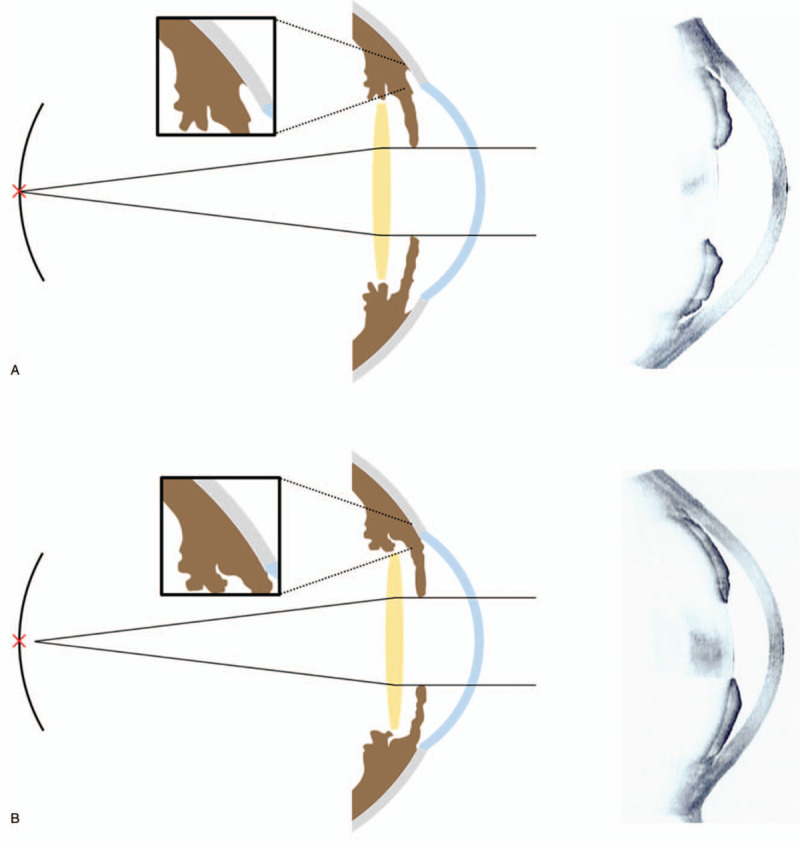
Possible mechanisms of refractive errors after cataract surgery in eyes with primary angle-closure with or without peripheral anterior synechiae (PAS) and representative images of anterior segment optical coherence tomography. (A) Eyes without PAS. (B) Eyes with PAS. Anterior chamber deepening was limited by PAS, consequently myopic shift could occur.

The reasons for the greater MAE and more myopic shift in eyes with PAS are unclear. A number of factors may explain this result. First, the presence of PAS may be evidence of the structural difference between the 2 groups. Prolonged apposition and repeated angle-closure attacks may lead to the development of PAS. Because there was no significant difference in age, AL, ACD, and mean keratometry between the two groups, eyes with PAS may have other structural abnormalities such as zonular loosening or a larger intracapsular bag to account for the difference. Unstable IOL positions (tilting or decentralization) due to large intracapsular bags or loose zonules may have induced more refractive error in eyes with PAS.^[8]^ Song et al reported that increased choroidal thickness was associated with a significant myopic shift after cataract surgery in PACD.^[23]^ Recently, there have been several studies on increasing choroid thickness in PACD,^[24–28]^ and these results indicate that the choroid is another structure involved in the pathogenesis of PACD. The presence of PAS might be the result of these various anatomical risk factors and further investigation is needed to prove their relationship. Second, the deepening of the anterior chamber after cataract surgery may have differed between the 2 groups. Lin et al quantified the effect of laser peripheral iridotomy (LPI) on angle widening in PACD with and without PAS.^[29]^ They found that the changes in anterior chamber angle after LPI were inversely correlated with the presence of PAS, and the parameters of ultrasound biomicroscopy did not change in quadrants with PAS. Although the effects of LPI and cataract extraction on the anterior chamber were not expected to be identical, deepening of the ACD after cataract surgery may have been affected by the presence of PAS. The posterior shifting of the IOL plane may have been limited by the presence of PAS; such limitation of posterior shifting of the IOL plane may have explained our observation of more myopic shift in eyes with PAS compared with eyes without PAS (Fig. 1). Consistent with this possibility, Yoo et al compared ultrasound biomicroscopy findings between eyes with PAS and without PAS, and reported that the trabecular-ciliary process distance was shorter in PACD eyes with PAS than in those without PAS,^[30]^ suggesting that anterior placement of the ciliary process may have played a role in the development of PAS.

Postoperative changes in anterior segment anatomy after phacoemulsification with IOL implantation may be an impediment to achieving consistent and precise refractive outcomes in PACD. The changes in cornea–iris–IOL relationships after cataract surgery are complicated and the position of the iris and IOL after surgery change differently according to AL.^[31]^ Although further studies are necessary to evaluate the effect of PAS on refractive outcomes associated with changes of anterior segment anatomy after surgery, our results suggest that the presence of PAS may be another factor affecting the outcomes of cataract surgery in PACD. Indaram et al reported 3 cases of myopic surprise after cataract surgery in plateau iris configuration patients.^[32]^ These cases also suggest that the angle configuration have an impact on the postoperative IOL position.

Notably, the MAEs or predicted refractive errors were not statistically different depending on the extent of PAS in the subanalyses, although the MAE of subgroup 2 (PAS ≥180°) tended to be larger than those of subgroup 1 (PAS <180°), and a more myopic shift was also found in subgroup 2. However, the sample size of the current study was too small to identify subtle differences in refractive error after cataract surgery between the 2 subgroups.

The present study had several limitations. First, the retrospective nature of this study may have introduced biases. Although we hypothesized that the limitation of posterior shifting of the IOL plane may have been the cause of the myopic shift in eyes with PAS, there was no objective evidence to support this possibility. Second, the timing of refractive error measurement after surgery was not identical among the study patients. However, several studies have reported that the refractive value stabilizes within 2 weeks after uncomplicated cataract surgery.^[33–36]^ Third, postoperative changes of PAS status or extent were not evaluated. Some investigators have reported a reduction in PAS after phacoemulsification in PACD.^[37,38]^ However, the amount of PAS change after phacoemulsification was different depending on the extent of the preoperative PAS,^[37]^ and the effects of postoperative PAS on refractive errors were not investigated. The present study suggested that further studies on postoperative gonioscopic findings and the effects of postoperative PAS on refractive errors are warranted to validate our hypotheses. Fourth, the variability of the CCC size may have affected the refractive outcomes after phacoemulsification. Nanavaty et al reported that the size of the CCC and the area of anterior capsule-IOL overlap influenced the IOL position.^[39]^ A larger CCC may lead to decentralization of the IOL, and a smaller CCC can increase the risk of anterior capsule fibrosis, which can lead to anterior capsular phimosis. However, it is not possible to create a constant CCC size or shape using the conventional manual method, especially in PACD eyes with loose zonules. In our study, the surgeon's goal was to create a CCC slightly smaller than the IOL optic margin; also, we excluded cases with anterior capsular phimosis after surgery to avoid bias. Fifth, the intervals between gonioscopy and cataract surgery were not controlled. PAS is not a stationary condition. Choi et al reported progression of PAS even after successful LPI, which may have led to the classification of eyes with PAS as eyes without PAS at the time of the cataract surgery.^[40]^ Sixth, the examination of PAS with gonioscopy was subjective and could have varied between observations. However, gonioscopy is still the gold standard to evaluate PAS, and it was performed by a single experienced investigator. Finally, suturing of the corneal wound may have affected refractive outcomes. However, we believe the effect of a corneal suture on the refractive outcomes was minimal because the corneal suture was removed as early as 1 week after surgery.

In conclusion, our study suggests that the IOL power prediction can be less accurate in PACD eyes with PAS compared with eyes without PAS. The presence or absence of PAS may influence the postoperative refractive outcomes in PACD patients; thus, it should be considered at the time of cataract surgery in these eyes. However, a further prospective study is needed to better assess the effects of the PAS on refractive outcomes after cataract surgery.

## Author contributions

**Conceptualization:** Tae-Eun Lee, Chungkwon Yoo, Yong Yeon Kim.

**Data curation:** Tae-Eun Lee, Chungkwon Yoo, Yong Yeon Kim.

**Formal analysis:** Tae-Eun Lee, Chungkwon Yoo.

**Investigation:** Tae-Eun Lee.

**Methodology:** Tae-Eun Lee, Chungkwon Yoo, Yong Yeon Kim.

**Project administration:** Tae-Eun Lee.

**Resources:** Tae-Eun Lee.

**Supervision:** Chungkwon Yoo, Yong Yeon Kim.

**Validation:** Chungkwon Yoo.

**Visualization:** Tae-Eun Lee.

**Writing – original draft:** Tae-Eun Lee, Chungkwon Yoo.

**Writing – review and editing:** Chungkwon Yoo.
